# Nutrigenomics of Natural Antioxidants in Broilers

**DOI:** 10.3390/antiox13030270

**Published:** 2024-02-22

**Authors:** Ioanna Kouvedaki, Athanasios C. Pappas, Peter F. Surai, Evangelos Zoidis

**Affiliations:** 1 Laboratory of Nutritional Physiology and Feeding, Department of Animal Science, Agricultural University of Athens, 75 Iera Odos, 11855 Athens, Greece; stud218033@aua.gr (I.K.); apappas@aua.gr (A.C.P.); 2 Vitagene and Health Research Centre, Bristol BS4 2RS, UK; psurai@feedfood.co.uk; 3 Faculty of Veterinary Medicine, Trakia University, 6000 Stara Zagora, Bulgaria; 4 Faculty of Agricultural and Environmental Sciences, Szent Istvan University, H-2103 Gödöllo, Hungary

**Keywords:** broilers, gene expression, natural antioxidants, oxidative stress, redox homeostasis, Nrf2, NF-κB, vitagenes

## Abstract

The broiler industry supplies high-quality animal protein to the world. The ban of antibiotics as growth promoters has opened the way for plenty of phytochemicals and antioxidants to be explored. This study summarizes the use of natural antioxidants in a broiler diet as a way through which to deal with stressors, as well as their effects on the expression of various genes. The transcriptional factors and genes involved in the regulation of redox homeostasis are described and emphasis is placed on nuclear factor erythroid 2-related factor 2 and nuclear factor kappa B. Sources such as fruits, vegetables, spices, mushrooms, and algae contain numerous natural antioxidant compounds. The antioxidant activity of these compounds has also been confirmed at the genome level. This study focuses on the regulation of oxidative stress-related genes, as well as on genes that regulate the inflammatory response, apoptosis, response to heat stress, lipid metabolism, and the intestinal barrier status. The natural compounds presented include, but are not limited to, the following: rutin, lycopene, magnolol, genistein, hesperidin, naringin, quercetin, curcumin, bisdemethoxycurcumin, resveratrol, astaxanthin, squalene, pterostilbene, protocatechuic acid, taraxasterol, myricetin, and proanthocyanidins. Several studies have revealed a dose-dependent action. Future studies should focus on the role of phytogenic compounds as antibiotic alternatives in relation to gut microbiota and their role in eubiosis.

## 1. Introduction

The challenge of feeding 9 billion people, by 2050, will determine the future of humanity [1]. Taking into account that the global population will continue to grow, the world is now facing a new challenge: food security. There is a competition for food resources on the ground, where a large percentage of humans, unfortunately, still do not have access to sufficient dietary protein and energy, let alone those that suffer from some form of micronutrient malnourishment [2]. Under this scope, there is a pressing need to tackle food security challenges under a dynamic multidimensional management of food–feed competition and environmental sustainability where animal protein is concerned. Worldwide, the leading meat production sector is the poultry industry as it efficiently supplies high-quality animal protein to the world. The meat poultry industry is dominated by the chicken meat industry, which is also known as the broiler industry [3]. At a global scale, the poultry sector is expected to continuously grow at a compound annual growth rate (CAGR) of 6.8% by 2030 [4].

A challenge facing the poultry industry is related to the spread of pathogens within commercial farms and, consequently, its high dependence on antibiotics and other pharmaceuticals. Although the inclusion of antibiotics at sub-therapeutical levels in broiler diets has proven to be an efficient strategy through which to suppress the pathogenic bacteria in the gut and enhance animal performance, their usage as growth promoters has been banned in Europe due to concerns regarding the consequences of antibiotic resistance on human health. Under this context, plenty of phytochemicals and antioxidants are being explored in broiler diets.

Broilers, as is the case for all aerobic living organisms, are capable of generating ROS (reactive oxygen species) to regulate important physiological functions while keeping them at low levels to maintain redox balance. However, there are many unfavorable conditions and challenges, either of endogenous (related to broiler) or exogenous (related to farm conditions) origin, which can disturb this balance through the overproduction of ROS at levels that the endogenous antioxidant system cannot cope with. As a result, lipids, proteins, DNA, and carbohydrates may be damaged, thereby inducing the organism’s inability to effectively absorb essential nutrients and thus leading to deteriorating conditions. Supplementation with natural antioxidants can also enhance its function by affecting gene expression and regulation, as well as apoptosis and signal transduction [5]. The aim of the present work was to summarize the use of natural antioxidants in the diet of broilers as a way of dealing with the problems caused by various stressors and their effects on the expression of various genes, thereby providing to the reader a summary of the published work, their limitations, and future research perspectives.

## 2. Data Sourcing and Inclusion Criteria

The present study involved a review that encompassed numerous studies with the objective of showcasing the overall positive effect of the dietary administration of natural antioxidants on the gene expression of broilers, specifically in the regulation of oxidative stress, inflammatory response, metabolism, and the intestinal barrier (either alone or in combination with external stress factors).

Possible studies eligible for inclusion in this review were identified through a search of databases such as Google Scholar, PubMed, Science Direct, Scopus, and Research Gate.

The search utilized indices involving combinations, appropriate truncations, and variations of the terms ‘natural antioxidant’, ‘broiler’, ‘dietary’, ‘supplementation’, ‘gene expression’, ‘oxidative stress’, ‘inflammation’, ‘metabolism’, ‘transcriptional factor’, ‘transcriptome’, ‘Nrf2’, ‘NF-κB’, ‘lipid metabolism’, ‘redox status’, and ‘antioxidant status’, as well as any plurals thereof. The analysis involved the assessment of published studies in English, extending up to and including the publication year 2019. Experiments were conducted on broilers that were receiving a dietary supplementation of any kind of natural antioxidant on a daily basis throughout the examined experimental period. Permissible experimental designs included controlled randomized feeding trials for multiple groups of broilers subjected to varying doses of natural antioxidants. Out of an initial database of 117 articles that covered diverse aspects of natural antioxidants and their effect on broiler gene expression, there were 29 feeding trials—involving a total of 8482 chickens across 116 treatment and control groups—identified for inclusion in this study. The majority of the experiments featured multiple treatment groups alongside a single control group.

### Range of Interest, Data Extraction, and the Dataset

The scope of interest in this study was limited to the exclusion of trials that administered the natural sources of natural antioxidants, such as leaves, seeds, roots, or the by-products from a processing industry. This exclusion was due to the lack of specified information regarding the varied antioxidants within these sources and their respective concentrations. Hence, a comprehensive evaluation of the direct impact of each individual natural antioxidant was impeded. This study involved exploring the utilization of high-purity natural commercialized antioxidants and their impact on the mRNA expression levels of genes associated with the oxidative stress response, inflammatory response, apoptosis, metabolism, and the intestinal barrier in broilers. Additionally, studies involving the administration of Se-enriched yeast were included; furthermore, as the concentration in each treatment was clearly specified, their effect could thus be assessed.

Various data points were recorded and employed in this study. The components of the dataset were as follows. The natural antioxidant type in each study; the dosage administered in each treatment; its impact on the regulation (up or downregulation) of genes associated with the oxidative stress response, inflammatory response, apoptosis, metabolism, and the intestinal barrier; and the principal conclusion drawn in each study were all examined. Studies that utilized natural commercialized antioxidants or Se-enriched yeast—whereby their impact on growth performance, antioxidant activity/capacity, intestine morphology/histology, meat quality, serum biochemical indexes, fatty acid profiles, etc. were detailed—were identified. However, those that failed to mention the expressed levels of related genes were subsequently excluded. Furthermore, the studies were categorized into two main groups based on whether the broilers were exposed to external stressors or not. The most prevalent stress factors identified included heat stress, lipopolysaccharide (LPS)-induced stress, mycotoxin-induced stress, and diquat-induced oxidative stress.

## 3. Oxidative Stress

### 3.1. The Endogenous Antioxidant System

In recent decades, it has become apparent that the excessive production of free radicals resulting in oxidative stress and redox imbalance constitutes the primary causative factors for the detrimental consequences of stress in poultry. Oxidative stress is characterized by an imbalance between oxidants and antioxidants. It can be further delineated into ‘oxidative eustress’ at low levels and ‘oxidative distress’ at high levels. At low levels, ROS, which is generated as the by-products of aerobic metabolism, interact with specific targets and play a significant role in redox signaling. They are responsible for stress adaptation, homeostasis, and the maintenance of health. However, exposure to high levels of ROS can lead to biomolecule damage, thus causing oxidative distress—which, in turn, disrupts redox signaling. This disruption can have a profound impact on the productive and reproductive performance of poultry. Throughout the course of evolution, integrated antioxidant defense systems were developed in poultry. These defense mechanisms are designed to regulate the production of free radicals and uphold the balance between antioxidants and prooxidants in the redox environment [5,6,7].

It has been suggested that the endogenous antioxidant system operates on three principal levels, which comprise natural fat-soluble antioxidants such as vitamin E, carotenoids, and ubiquinones; water-soluble antioxidants like ascorbic acid, uric acid, carnitine, and taurine; and a collection of antioxidant enzymes, namely glutathione peroxidase (GPx), catalase (CAT), and superoxide dismutase (SOD). Additionally, the thiol redox system—which encompasses the glutathione system (comprising glutathione, glutathione reductase, glutaredoxin, and glutathione peroxidase) and the thioredoxin system (comprising thioredoxin, thioredoxin reductase, thioredoxin peroxidase such as peroxiredoxins, and sulfiredoxin)—is integral to this comprehensive antioxidant defense mechanism [5].

The first level of defense, which is tasked with averting radical formation, sustains the redox balance, as well as participates in cell signaling (which encompasses essential enzymes like SOD, GPx, CAT, and metal-binding proteins, but also the thioredoxin system, glutathione system, vitagenes, and transcription factors such as Nrf2 (nuclear factor erythroid 2-related factor 2), NF-κB (nuclear factor kappa B), and HSF (heat shock factor)). Since the superoxide radical (O_2_^•^) is the primary radical produced under normal cellular conditions, SOD is regarded as a key component in the first level of defense. Through its involvement, O_2_^•^ is transformed into hydroperoxide (H_2_O_2_). Recognized as toxic to cells, H_2_O_2_ undergoes a detoxification that is facilitated by a range of enzymes, including catalase, GPx, and peroxiredoxins [5].

Despite its best efforts, the first level of defense proves insufficient in completely preventing the formation of free radicals. This inadequacy results in the release of radicals that are capable of initiating lipid peroxidation and causing damage to DNA and proteins. Consequently, a second level of defense was established, which comprises chain-breaking antioxidants such as vitamin E, ubiquinol, carotenoids, ascorbic acid, uric acid, carnitine, and taurine, as well as various other antioxidants. These compounds work to inhibit peroxidation by minimizing the chain length of the propagation reaction. Vitamin E is recognized as the most effective natural free radical scavenger; however, it can lead to the production of hydroperoxides that are toxic to the cell. Thus, vitamin E and GPx operate collaboratively, thereby offering a comprehensive and effective antioxidant defense. Coenzyme Q, also recognized as ubiquinone, actively participates in shielding biological molecules, including lipids, proteins, and DNA, from oxidative damage. It accomplishes this by neutralizing free radicals, replenishing other antioxidants (such as vitamins E and C), and overseeing the maintenance of mitochondrial integrity. Additionally, the second level of antioxidant defense involves the active participation of the glutathione and thioredoxin systems. Glutathione (GSH), an abundant non-protein thiol in avian cells, stands as one of the most crucial non-enzymatic antioxidants in poultry. It actively contributes to the maintenance of redox balance and signaling, as well as to the regulation of transcription factors and gene expression [5].

Nevertheless, even the second level of antioxidant defense proves inadequate in preventing the detrimental impact of reactive oxygen species (ROS) on lipids, proteins, and DNA. In such instances, the third level of defense relies on systems that are designed to either eliminate or repair damaged molecules. This level encompasses lipolytic enzymes (lipases) and proteolytic enzymes (peptidases or proteases), as well as various other enzymes (DNA repair enzymes, ligases, nucleases, polymerases, proteinases, phospholipases, various transferases, etc.). Additionally, protein chaperones, including heat shock proteins (HSPs), play a crucial role in this third level of defense [5].

Antioxidants function collectively within the body, and they constitute an integrated system that is designed to mitigate oxidative stress. However, these adaptive mechanisms exhibit limited efficacy. Once the production of free radicals surpasses the antioxidant system’s capacity for neutralization, lipid peroxidation ensues, thus resulting in damage to unsaturated lipids in cell membranes, amino acids in proteins, and nucleotides in DNA. Consequently, membrane and cell integrity become compromised [5].

### 3.2. Oxidative Stress and Gene Expression

**Figure 1:**
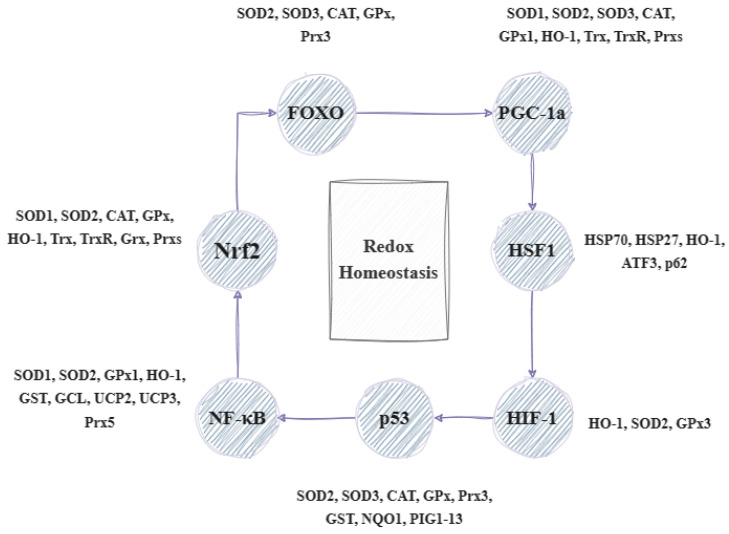
Illustration of the transcriptional factors and genes involved in the regulation of redox homeostasis (based on [5] with modifications). FOXO: forkhead box O transcription factor, PGC-1a: peroxisome proliferator-activated receptor-gamma coactivator, HSF1: heat shock factor 1; HIF-1: hypoxia-inducible factor 1; p53: cellular tumor antigen 53; NF-kB: nuclear factor kappa B; Nrf2: nuclear respiratory factor 2; SOD1–3: superoxide dismutase 1–3; CAT: catalase; GPx: glutathione peroxidase; Prx3,5: peroxiredoxin 3, 5; HO-1: heme oxygenase-1; Trx: thioredoxin; TrxR: thioredoxin reductase; Grx: glutaredoxin; GST: glutathione S-transferase; GCL: glutamate–cysteine ligase; UCP2,3: uncoupling protein 1, 3; NQO1: NAD(P)H:quinone oxidoreductase 1; PIG1–13: p53-inducible genes 1–13; and ATF3: activating transcription factor 3.

Under oxidative stress, the body initiates a response process that includes the activation of the gene expression of the defense systems. Mitogen-activated protein kinases (MAPK) are important in the cell’s response to the hormones, cytokines, and signals generated by oxidative stress. Apoptosis signal-regulating kinase 1 (ASK1), which is activated in various stressful situations, causes the upregulation of MAPK. Several proteins appear to influence the action of ASK1. For example, thioredoxin can lead to its direct inhibition as its reduced form can bind to ASK1, thereby inhibiting its dimerization and activation. During oxidative stress, thioredoxin is oxidized, which results in its uncoupling from ASK1, thus enabling its activation and involvement in the p38 signal transduction and leading to non-apoptotic effects. ROS can either stimulate protein kinases G (PKG), -A (PKA), and -C (PKC), which—in turn—can regulate MAPK activation or have an immediate impact on activating the MAPK pathway as they can act directly on MAPK phosphatases (MKPs). The MAPK subfamilies include kinases such as ERK (extracellular regulated kinases, i.e., ERK1/2, ERK3/4, ERK5, and ERK7/ERK8); p38; JNK (c-Jun N-terminal kinase); and NLK (Nemo-like kinase) [8,9]. The ERK pathway is mainly associated with regulating cell proliferation, while that of p38 and JNK is related to stress. For this reason, the latter two are grouped together and referred to as stress-activated protein kinases (SAPK) [10]. The most important effect of ROS on MAPKs involves the regulation of transcription factors that control the expression of many protective genes, which contributes to the termination of impaired cell division and leads to apoptosis [11]. Such factors are Nrf2 (nuclear factor erythroid 2-related factor 2) and NF-κB (nuclear factor kappa B). The transcription factor Nrf2 is responsible for the activation of genes that lead to the synthesis of protective antioxidant molecules, while NF-κB activates the genes involved in inflammatory, immune, and acute phase responses. Oxidative stress can cause damage to various molecules in the body. In response to this, other reactions, such as HSR (heat shock response), UPR (unfolded protein response), HIR (hypoxia-induced response), DNA damage response, etc., are triggered to regulate the corresponding transcription factors like HSF1 (heat shock factor 1), FOXO (forkhead box O), HIF (hypoxia-inducible factor), p53 (cellular tumor antigen 53), etc. (as shown in Figure 1).

**Figure 2:**
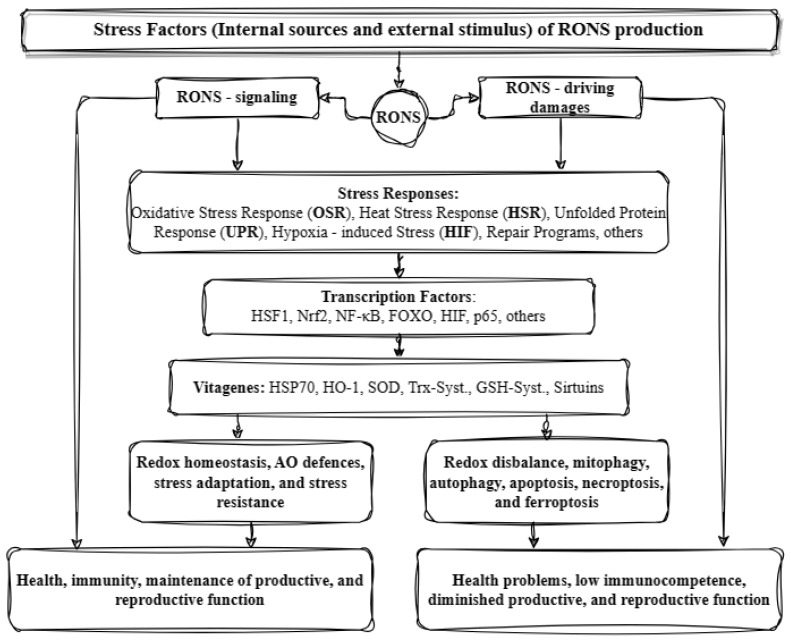
Illustration of the antioxidant defense system in animals (based on [6] with modifications). RONS: reactive oxygen and nitrogen species; HSF1: heat shock factor 1; Nrf2: nuclear respiratory factor 2; NF-kB: nuclear factor kappa B; FOXO: forkhead box O transcription factor; HIF: hypoxia-inducible factor; p65: cellular tumor antigen 65; HSPs: heat shock proteins, HO-1: heme oxygenase 1; SOD: superoxide dismutase; Trx-Syst.: thioredoxin system; GSH-Syst.: glutathione system; and AO: antioxidant.

These factors lead to the regulation of several genes that help in stress sensing and maintaining redox homeostasis. These genes are known as vitagenes and were first described in 1998 to signify the body’s efficient functioning in repairing and maintaining itself through a variety of processes regulated by these genes. The term ‘vitagenes’ was later coined by the medical sciences to refer to a group of genes that are responsible for upholding cellular homeostasis during periods of stress. This group encompasses several gene families such as heat shock proteins (HSPs), the thioredoxin system, sirtuins, and SOD (superoxide dismutase). HSPs comprise HO-1 (heme oxygenase-1) and HSP70 (heat shock protein 70), while SODs include SOD1, SOD2, and SOD3. The thioredoxin and glutathione system involves Trx (thioredoxin), TrxR (thioredoxin reductase), Prx (peroxiredoxin), Srx (sulfiredoxin), and GSH (glutathione), as well as GR (glutathione reductase), GPx (glutathione peroxidase), and Grx (glutaredoxin) [12]. Lastly, sirtuins (SIRTs) consist of SIRT1, SIRT2, SIRT3, SIRT4, SIRT5, SIRT6, and SIRT7. The products of these genes can detect various forms of stress and regulate the production of different protective molecules. Sirtuins can influence the expression and activity of many factors. For instance, SIRT1 can combat oxidative stress by regulating transcription factors, such as PPAR (peroxisome proliferator-activated receptor), NRF (nuclear respiratory factor), and TFAM (mitochondrial transcription factor A). Moreover, it aids in enhancing the expression of SOD and GPX while also triggering the FOXO/MnSOD signaling pathway by regulating the acetylation of the FOXO factor. This ultimately results in an increase in the expression of MnSOD and catalase, which can effectively counteract oxidative stress. It can also regulate the inflammatory response by acting on NF-κB and thus affecting the transcription of genes such as IL-1 (interleukin-1), TNF-α (tumor necrosis factor α), IL-8 (interleukin-8), and IL-6 (interleukin-6). Similarly, vitagenes can be affected by various transcription factors. The activation of factors such as HSF and FOXO results in the increased production of HSP70 and sirtuins. Similarly, the activation of Nrf2 leads to an increased synthesis of SOD, HO-1, and the compounds in the glutathione and thioredoxin systems. Certain vitagenes can be activated without the involvement of transcription factors. When the body’s antioxidant defense system is unable to forestall the damage caused by ROS, a range of mechanisms, including mitophagy, autophagy, apoptosis, necroptosis, and ferroptosis, are implemented (Figure 2) [5,6,13,14].

The process of apoptosis through the mitochondrial pathway is regulated by a group of proteins known as the B-cell lymphoma 2 (Bcl-2) family. With over 30 proteins in this family, each contain up to four conserved Bcl-2 homology domains (BH1–4) that are essential for their function, with BH3 being the death domain. This family of proteins can be divided into two categories: anti-apoptotic survival proteins and pro-apoptotic proteins. The anti-apoptotic proteins include Bcl-2, Bcl-XL, Bcl-W, Bcl-B, A1, and Mcl-1. Conversely, the pre-apoptotic proteins include Bax, Bak, Bok, Bok, Bad, Bik, Bmf, Hrk, SOUL, Bid, Bim, Puma, and Noxa. Each cell comprises an assortment of proteins from both categories, thereby indicating their interdependence and functional significance. The regulation of these proteins is crucial for cell survival. Apoptosis can be triggered by the activation of TNF-R (tumor necrosis factor receptors). Studies have shown that SIRT1 may impact the transcription of genes such as IAP (the inhibitor of apoptosis protein), Bcl-2, and TNF-R. Autophagy, on the other hand, is regulated by autophagy-related genes (Atgs) and the kinase pathway known as the mammalian target of rapamycin (mTOR). Autophagy, a process that leads to the degradation of cellular components, is inhibited by the mTOR kinase when there are enough nutrients and growth factors present. Death receptors such as TNF-R1 and Fas (apoptosis-mediating surface antigen), toll-like receptors, viruses, and DNA damage can trigger necroptosis [14,15].

### 3.3. Transcription Factor Nrf2

Nuclear factor erythroid 2-related factor 2 is a vital transcription factor responsible for regulating antioxidant and detoxifying enzymes through a domain referred to as ARE (antioxidant response element) or EpRE (electrophile responsive element) [16]. The function of Nrf2 is controlled by Keap1, a cytosolic repressor protein also referred to as the cytosolic repressor protein Kelch-like ECH-associated protein 1. Initially, it was believed that Keap1 confines Nrf2 in the cytoplasm and releases it under stress conditions, thus allowing Nrf2 to travel to the nucleus and regulate gene expression. However, further research has demonstrated that Nrf2 is inherently unstable and undergoes degradation by ubiquitin. Therefore, it has been proposed that Keap1 actively promotes the ubiquitination of Nrf2, thereby resulting in its breakdown and preventing its translocation to the nucleus. Follow-up experiments have confirmed this hypothesis [17,18]. Nrf2 is made up of seven conserved regions that are present in different species, which are known as Neh1–7 domains. Neh1 acts as a site for small protein heterodimerization and DNA binding. Neh4 and Neh5 are critical areas for Nrf2 post-activation, while Neh3, situated at the C-terminal end, is considered essential. The Neh2 domain’s N-terminal end is linked to Keap1, which regulates Nrf2 negatively by altering its subcellular structure and increasing its degradation rate. Neh6 is pivotal in controlling Nrf2 degradation in the nucleus. Moreover, Neh7, in conjunction with Neh6, facilitates the additional post-translational negative regulation of Nrf2. This regulation is not reliant on redox, unlike the one mediated by Neh2 and Keap1 [19,20,21,22,23]. The Keap1-Nrf2 pathway is a crucial regulator of the antioxidant system. When ROS are present, Keap1’s cysteine thiols may undergo oxidation, which alters its bonding with Nrf2. Consequently, Nrf2 migrates to the nucleus and pairs with small MAF proteins (musculoaponeurotic fibrosarcoma proteins) forming Nrf2-MAF heterodimers. These heterodimers have the ability to recognize and attach to a specific DNA sequence called ARE/EpRE. Once Nrf2 binds to this region, it activates the transcription of more than 250 genes that contain this sequence in their regulatory regions [24,25,26,27]. During the late 1980s, researchers conducted initial investigations into the presence of AREs, with a primary focus on xenobiotic metabolism. Through these studies, it was revealed that specific compounds could induce xenobiotic metabolism by synthesizing phase I and II enzymes.

**Figure 3:**
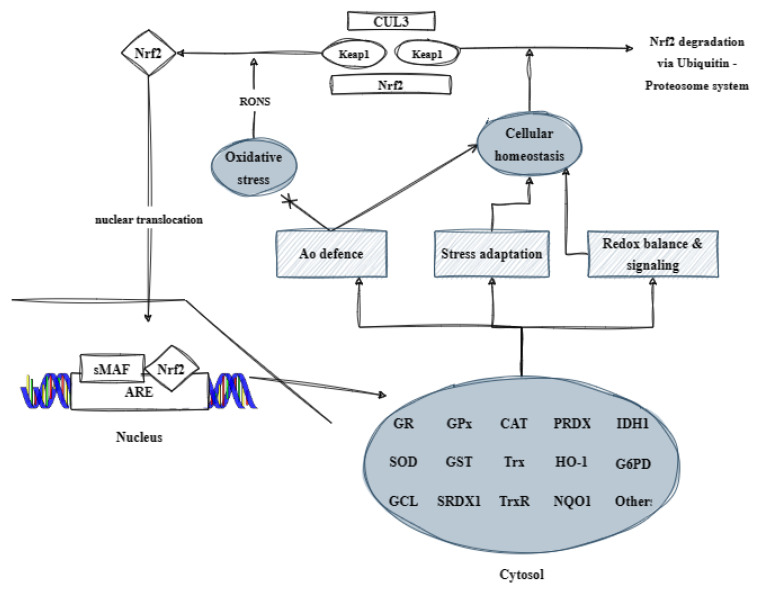
Illustration of the contribution of the transcription factor Nrf2 to the organism’s antioxidant defense (based on [5] with modifications). CUL3: Cullin 3; Keap1: kelch-like ECH-associated protein 1; RONS: reactive oxygen and nitrogen species; Nrf2: nuclear respiratory factor 2; Ao: antioxidant; sMAF: small musculoaponeurotic fibrosarcoma protein; ARE: antioxidant response element; GR: glutathione reductase; GPx: glutathione peroxidase; CAT: catalase; PRDX: peroxiredoxin; IDH1: histidinol dehydrogenase 1; SOD: superoxide dismutase; GST: glutathione S-transferase; Trx: thioredoxin; HO-1: heme oxygenase; G6PD: glucose-6-phosphate dehydrogenase; GCL: glutamate cysteine ligase; SRDX1: EAR motif-based artificial transcriptional repression domain 1; TrxR: thioredoxin reductase; and NQO1: NAD(P)H quinone dehydrogenase 1.

Other compounds, on the other hand, only synthesize phase II enzymes, such as glutathione S-transferase (GST), NADPH quinone oxidoreductase (NQO), etc. [28]. The activation of Nrf2 can also occur through the phosphorylation of specific serine and/or tyrosine residues on Keap1. This leads to the separation of Nrf2 and its transfer to the nucleus, where it binds to MAF and ARE, and regulates the expression of protective molecules. These include antioxidant enzymes such as SOD, GPx, CAT, GR, GCL, Trx, TrxR, and PRDX1; detoxification enzymes such as HO-1, NQO1, and GST; GSH-related proteins like γ-GCS (γ-glutamylcysteine synthetase); NADPH-producing enzymes; and various others. By controlling the expression of these proteins, Nrf2 helps to prevent the damage to the body caused by oxidative stress and inflammation (Figure 3) [5].

Furthermore, it has been reported that Nrf2 also regulates the expression of the genes that play a crucial role in lipid synthesis and uptake. These genes include SREBPs (sterol regulatory element-binding proteins), FAS (fatty acid synthase), SCD (stearoyl-CoA desaturase), and PPAR (peroxisome proliferator-activated receptor) [29].

### 3.4. Transcription Factor NF-κB

Nuclear factor kappa B is a transcription factor that can be activated in lymphocytes and plays a crucial role in the body’s inflammatory response. The inflammatory response is a natural process that involves the migration of blood cells and proteins to the site of injury, infection, trauma, or other stimuli that can initiate an immune response. This process is closely linked to oxidative stress. Tissue damage triggers the production of prostaglandins (PGs), leukotrienes (LTs), interleukins (ILs), cytokines, hydrolytic enzymes, and ROS [30]. This response can be triggered by various factors, such as oxidative stress, TNF-α, bacterial lipopolysaccharide (LPS), viruses, UV, and γ-rays, and it is known to regulate the expression of various genes, including pro-inflammatory cytokines, leukocyte adhesion molecules, acute phase proteins, and antimicrobial peptides. Within mammals and birds, the NF-κB protein complex is composed of five subunits, each of which possess the capacity to bind to DNA. These are p50 (or NF-κB1), p52 (or NF-κB2), p65 (or RelA), c-Rel, and RelB, which are encoded by the genes nfkb1, nfkb2, rela, crel, and relb, respectively. The NF-κB family comprises numerous transcription factors possessing a Rel homology domain (RHD) at their N-terminal end, which enables the formation of homo- and heterodimers. The NF-κB dimers are capable of binding to specific DNA regions known as “κB sites” to regulate gene expression. These sites are present in the enhancers and promoters of several genes. In addition, p65, c-Rel, and RelB possess a transcription activation domain (TAD) at their C-terminal end, which aids in the positive regulation of gene expression. On the other hand, subunits that lack the TAD domain (i.e., p50 and p52) can activate transcription by forming heterodimers with p65, c-Rel, or RelB, or by enlisting other proteins with TAD. Homodimers are unable to elicit transcription via DNA binding and instead inhibit the transcription process. The regulatory mechanisms for NF-κB exhibit similarities with those of Nrf2. For instance, the protein exists in an inactive state in the cytoplasm and is bound by inhibitory proteins known as IκB (e.g., IκBα, IκBβ, IκBγ, IκBδ, IκBε, IκBζ, p100, p105, Bcl-3, and IκBns). Under oxidative stress, these two factors compete to coordinate an appropriate response. In the presence of cytokines and other stressors, the activation of NF-κB transpires. Specifically, when a dimer of NF-κB enters the nucleus, the IκB bound to it subsequently undergoes phosphorylation at specific serine residues through the IκB kinase (IKK) complex. The phosphorylation process is a significant event that initiates the NF-κB signaling pathway. This, in turn, leads to the expression of a diverse set of genes that play a crucial role in immune and inflammatory responses. The IKK complex is made up of two active kinases, namely IKKα (IKK1) and IKKβ (IKK2), along with a regulatory scaffolding protein known as NEMO (IKKγ) [31,32,33,34]. Activators of the IKK complex include mitogen-activated protein kinases (MAP3Ks) such as MEKK1, MEKK3, and TAK1 [35]. NF-κB and Nrf2 have an antagonistic relationship, where one inhibits the activation of the other. Nrf2 can prevent the activation of NF-κB by increasing the antioxidant defense, thereby reducing ROS and preventing the degradation of IκBα, as well as inhibiting the transcription of proinflammatory genes. Similarly, Keap1 can also act as IKKβ and negatively regulate NF-κB by stabilizing IκBα, thereby leading to the inhibition of its activation. In contrast, NF-κB may impede the activation of Nrf2 by inducing the recruitment of a histone to the ARE domain, which—in turn—restrains the transcription of the corresponding genes. Nrf2 and p65 compete for the CBP-p300 complex, which is responsible for transferring an acetyl unit to the lysine residues of transcription factors. This enhances gene transcription. Generally, CBP (CREB binding protein) exhibits a preference for κB genes [5,36,37].

## 4. Effects of Natural Antioxidants on Gene Expression

Many natural antioxidants obtained through diet are effective in regulating various signaling pathways, transcription factors, and genes. For instance, flavonoids like anthocyanins [38], carotenoids such as lycopene [39,40,41], and astaxanthin [42] are known to influence the transcription of the genes related to the oxidative stress response and detoxification by activating Nrf2/ARE and NF-κB. Curcumin interacts with various intracellular systems like NF-κB, iNOS (inducible nitric oxide synthase), HIF-1, and several members of the vitagene family (e.g., HO-1). Quercetin, a flavonol found in berries, apples, cereals, grapes, etc., can increase the expression of Nrf2 mRNA by enhancing the antioxidant system [43,44,45,46]. Naringenin contributes to the suppression of NF-κB activation in macrophages, thereby leading to the inhibition of IL-33, TNF-α, IL-1β, and IL-6, while genistein can limit the expression of pro-inflammatory cytokines such as IL-1β and TNF-α by inactivating NF-κB [47]. Gallic acid, a phenolic compound found in berries, strawberries, various types of tea, and herbs, was found to increase the activity of antioxidant enzymes by regulating Nrf2/NF-κB mechanisms, thus increasing the expression of HO-1 and Nrf2 genes and decreasing that of NF-κB [48]. In general, tea polyphenols (EGCG: epigallocatechin-3-gallate, ECG: epicatechin-3-gallate, EGC: epigallocatechin, and EC: epicatechin) can affect the expression of Nrf2 and NF-κB transcription factors [49]. Supplementation with EGCG can effectively alleviate heat shock in poultry by regulating Nrf2/ARE and NF-κB systems [50]. Other flavonoids, such as resveratrol, and various chalcones can also activate Nrf2 by stimulating the gene expression of HO-1, NQO1, etc., which also reduces the inhibitory effect of NF-κB on antioxidants [51,52]. Furthermore, other polyphenolic compounds, including silymarin, are also shown to activate Nrf2 and suppress NF-κB in various in vitro and in vivo model systems [53]. In addition, taurine [54] and carnitine [5] are also important modulators of the Nrf2/NF-κB pathways under various stress conditions.

Another pathway that can be activated to adapt cells to stress is the sirtuin–FOXO pathway. SIRT1 can deacetylate many substrates, including several transcription factors such as FOXO-1, -3, and -4, and the p65 subunit of NF-κB (which inhibits NF-κB activity). Several phytochemical compounds, such as resveratrol, have been suggested to activate FOXO deacetylation via sirtuins, thereby increasing cellular stress tolerance [55]. Resveratrol indirectly activates SIRT1 through AMPK (AMP-activated protein kinase)-dependent mechanisms [56]. Phenolic compounds such as fisetin, quercetin, and curcumin can increase SIRT1 expression [14,57]. Polyphenols like kaempferol, luteolin, chrysin, and apigenin can inhibit the activity of MAPK and its various subfamilies [9]. Different types of compounds have different effects on the expression of Bcl-2, Bcl-XL, and Bax. For example, stilbenes and chalcones decrease the expression of Bcl-2, while flavonols and flavones increase the expression of Bax and decrease that of Bcl-2. Anthocyanins and lignans can decrease the expression of Bcl-2 and Bcl-XL, and coumarins can decrease the expression of Bcl-XL by increasing that of Bax [58]. Apigenin inhibits PPAR activity by activating Nrf2, which inhibits lipid metabolism and increases then number of oxidative stress genes [59]. An overabundance of these elements may be detrimental to cell function. As a result, researchers have been continuously exploring their effects and safety while devising innovative approaches to analyze them. Given that certain elements may have multiple functions; they may not be the most effective treatment for a given condition. Additionally, due to their lower potency, consistent administration is required to achieve the desired therapeutic outcome [55].

## 5. Supplementation of Natural Antioxidants in the Diet of Broilers and Their Effects on Gene Expression

The poultry farming sector dedicated to meat production stands as one of the predominant branches within global livestock farming. Nevertheless, it frequently encounters challenges, as any deviation from optimal internal and external conditions can lead to stress. In this context, there are mainly four categories of stressors: technological, environmental, nutritional, and internal. In the technological category, factors include increased stocking density, the management of chicks (weighing, grouping, and transportation), and the management of fertilized eggs (extended storage duration, transportation, inappropriate storage conditions, incorrect incubation regimes related mainly to temperature, humidity, and CO_2_ concentration). The main environmental stressors are inadequate temperature, poor ventilation (high levels of ammonia and CO_2_), increased dust levels, and insufficient lighting. Nutritional factors include the presence of mycotoxins in feed; oxidized fats; toxic metals such as lead (Pb), cadmium (Cd), and mercury (Hg); whether there is an unbalanced diet (deficiencies in nutrients and trace elements such as Se, Zn, Mn, Cu, and vitamin overdosing); poor water quality; and the extensive use of coccidiostats and other drugs through feed or water (i.e., the risk of reducing antioxidant absorption) [60,61]. Internal stressors include vaccinations, as well as bacterial and viral infections.

A significant majority of these stress-inducing factors are related to oxidative stress and the generation of ROS, thereby causing molecular and cellular alterations, as well as compromised performances and substantial economic losses [7,62]. The diminished capacity of the antioxidant system to cope with extensive ROS production can be ameliorated through the dietary supplementation of natural antioxidants. These compounds are acknowledged for their potential in influencing the function of transcription factors and the genes associated with an organism’s stress response. Notable among these natural antioxidants are carnitine, betaine, taurine, vitamin E, ascorbic acid, and Se, as well as a plethora of polyphenols and flavonoids that have been reported to successfully impact gene expression [5,63,64].

Dietary supplementation involving magnolol at concentrations of 200 and 300 mg/kg has been observed to elevate the levels of Nrf2, NQO1, HO-1, GST, GCLC (glutamate-cysteine ligase catalytic subunit), GCLM (glutamate cysteine ligase modifier subunit), and SOD, thereby enhancing both the antioxidant capacity and growth performance of broilers along with other positive effects on meat quality [65]. Furthermore, the administration of 30 mg/kg lycopene effectively has been found to activate the Keap1-Nrf2 signaling pathway, thereby regulating the expression of the genes associated with the enhancement of antioxidant activity in both the serum and liver of broilers [66].

Additionally, the affirmative impacts of numerous natural antioxidants on the regulation of genes linked to gut barrier function, inflammatory response, apoptosis, and lipid metabolism in poultry have been documented. The intestinal barrier comprises a constellation of constituents designed to impede the invasion of pathogens and toxins into the gastrointestinal tract. Among these are intercellular tight junctions (TJ), which are protein complexes consisting of proteins such as claudin, occludin, and JAM-A (junctional adhesion molecule-A), as well as intracellular plaque proteins, such as zonula occludens (ZOs). Phytochemical compounds such as resveratrol, naringenin, quercetin, etc. can play a significant role in the expression of these proteins. By extending beyond the modulation of NF-κB and Nrf2, these compounds can impact the intestinal barrier not only by enhancing the expression of TJ, but also by instigating the metabolic pathways for cytokine production [67]. Rutin, particularly at a dosage of 500 mg/kg, exhibits ameliorative effects on the intestinal barrier function, the immune response, and the antioxidant capacity of supplemented broilers. These effects have been attributed to the upregulation of the genes related to the intestinal barrier, the inhibition of NF-κB, and the activation of the Nrf2/HO-1 signaling pathway [68]. Furthermore, many flavonoid species (flavones, flavanones, flavonols, etc.) and other compounds have been identified to influence lipid metabolism and deposition by regulating the expression of genes such as PPARγ, PPARα, SREBP1, FAS, etc. [69]. Genistein has been reported to suppress fat deposition by upregulating the genes associated with lipogenesis (LXRα: liver X receptor-α, SREBP-1c, FAS, and ACC) and concurrently downregulating those linked to lipolysis (PPARα, ATGL: adipose triglyceride lipase, and CPT-I: carnitine palmitoyltransferase I), which are attributed to the activation of the AMPK-SIRT1/PGC-1a signaling pathway [70]. Curcumin downregulates the expression levels of the genes related to lipogenesis such as FAS, SREBP-1c, ACC (acetyl-CoA carboxylase), and ACLY (ATP-citrate lyase), while it also concurrently upregulates those responsible for lipolysis, including PPARα and CPT-I, thus indicating its effect on the reduction in abdominal fat deposition [71]. Hesperidin and naringin have been associated with the enhancement of oxidative stability in meat, which is achieved through the regulation of the GSR (glutathione-disulfide reductase) gene, while naringin also significantly increases the expression levels of PPARα and ACOX1 (acyl-CoA oxidase 1), thus positively affecting the FA (fatty acid) profiles in the breast meat and fat pad of broilers [72]. Rutin supplementation at concentrations of 200 or 400 mg/kg demonstrated a positive impact on the antioxidant activity and FA profiles orchestrated through the regulation of NRF2, CAT, and PPARG, as well as FADS1 (fatty acid desaturase-1), FAS, and ELOVL7 (fatty acid elongase 7) in breast muscle [73]. Varied concentrations of quercetin, as documented in the study by Wang M. et al. (2021), have been observed to contribute to the reduction in hepatic fat deposition in broilers, which is achieved through the activation of the AMPK/PPAR signaling pathway [74]. Furthermore, supplementation with 200–400 ppm quercetin, as suggested by Abdel-Latif et al. (2021), has proven effective in enhancing broiler development through the regulation of nutrient transporter genes that have important roles in nutrient metabolism like GLUT2 (glucose transporter 2), PEPT1 (peptide transporter 1), and FAS [75].

Currently, several studies have investigated the impact of these natural antioxidants in scenarios where broiler chickens were subjected to various stressors, including heat stress, lipopolysaccharide (LPS)-induced stress, or mycotoxin-induced stress. Under diquat-induced oxidative stress, broilers that were fed with a basal diet supplemented with squalene exhibited an enhanced oxidative status, one that was characterized by higher expression levels of Nrf2 and GPX1. Simultaneously, a significant mitigation of liver injury was achieved through the regulation of apoptosis-related genes (Bax and CASP3, i.e., caspase 3) [76]. Similarly, the administration of 400 mg/kg of pterostilbene to 21-day-old broiler chickens demonstrated efficacy in alleviating diquat-induced liver damage through the regulation of antioxidant-related genes (HO-1, SOD1, GSTA2, and γ-GCLc) and apoptosis-related genes (BCL2 and CASP3) [77]. Furthermore, when faced with an oxidative stress induced by oxidized oil, the supplementation of broilers with 800 ppm of quercetin displayed a capacity to ameliorate the oxidative stress, as evidenced by the upregulation of Nrf2, GCLM, CAT, SOD1, GPX2, GLRDX, TXN, and HO-1, as well as by the restoration of the redox balance while also strengthening the intestinal barrier via the upregulation of MUC2 (mucin 2) and claudin-2 [78]. Numerous studies have implicated high temperatures as a stress-inducing factor. Under heat stress, supplementation with microalgal astaxanthin was observed to influence the mRNA levels of various redox status-controlling genes (GPX1, GR, and SOD1); genes related to heat stress (HSP70 and HSTF1, i.e., heat shock transcription factor 1); genes related to the inflammatory response (JNK1 and TNF-α), and genes related to lipid metabolism (SREBP1 and DGAT2, i.e., diacylglycerol O-acyltransferase 2) [79]. Similarly, the detrimental effects of heat stress were mitigated through the supplementation of various doses of astaxanthin, which resulted in the downregulation of HSP27, HSP70, TNF-α, and IL-6. This led to an improvement in antioxidant status and enhanced broiler meat quality [80]. Furthermore, broilers challenged with high temperature and fed with 400 mg/kg of resveratrol exhibited higher muscle antioxidant capacity due to the activation of the Nrf2 signaling pathway and the regulation of the genes associated with antioxidant activity, which served to minimize the deterioration of meat quality [81]. Simultaneously, supplementation with 400 mg/kg of resveratrol demonstrated an improvement in intestinal antioxidant capacity through the activation of the Nrf2 signaling pathway, which led to the upregulation of critical factors like SOD1, GPX, and GST [82]. In another study, the supplementation with resveratrol demonstrated a significant decrease in the expression levels of NF-κB, HSP70, and p38 MAPK, thus indicating its potential in reducing the splenic inflammatory response and apoptosis in heat-stressed broilers [83]. To enhance resveratrol’s bioavailability, Kishawy et al. (2023) encapsuled this compound into liposomal nanoparticles and assessed its effect on broiler gene expression when under heat stress. Despite using concentrations lower than usual (i.e., 50, 100, and 150 mg/kg vs. 400–500 mg/kg), similar results were found. The groups that received Resv-Lipo NPs showed a restoration of the high expression of heat shock proteins and pro-inflammatory cytokines caused by high temperature. Moreover, they demonstrated a higher regulation of antioxidant-related genes (SOD, CAT, GSH-PX (glutathione peroxidase), Nrf2, and HO-1) and muscle-building genes (MyoD (myogenic differentiation 1) and mTOR), thus indicating a positive effect on mitigating heat stress and improving broiler performance [84]. Under heat stress conditions, intestinal villi disruptions and liver necrotic hepatocytes were observed while the villi integrity was significantly enhanced by a seleno-yeast treatment, thereby leading to improved layer performance in the heat-stressed layers [85]. Furthermore, metabolism-related signaling pathways (including PI3K: phosphatidylinositol 3-kinase; Akt: Akt kinase; Wnt: wingless-related integration site; CA: carbonic anhydrase; IGF1 receptor-: insulin-like growth factor 1 receptor; and MAPK signaling pathways) have been demonstrated to contribute to the regulation of organic Se on broiler liver metabolism and health [86].

Interesting findings have emerged from studies utilizing bacterial lipopolysaccharide (LPS) as a stress-inducing factor. In particular, a dietary supplementation with 40 mg/kg of lutein has demonstrated a notable enhancement in the LPS-induced jejunal mucosal barrier function, which was also found to be accompanied with a mitigation of inflammation through the, respective, regulation of occludin, claudin-1, and ZO-1, as well as IL-1β, IL-6, IL-4, and IL-10 [87]. Moreover, the damage induced by LPS in the jejunum and ileum of the broilers was partially alleviated with the supplementation of 400 mg/kg of resveratrol, as evidenced by the increased mRNA expression of claudin-5, occludin, and zonula occludens-1 (ZO-1), as well as the reduced expression of IL-1β and TNF-α [88]. Additionally, supplementation with 600 mg/kg of protocatechuic acid alleviated the damage in the intestinal barrier caused by LPS injection, while also concurrently improving the antioxidant status of the broilers, thereby regulating the respective expression levels of related genes like OCLN, ZO-1, JAM2, MUC2, CAT, SOD1, and GPx-1 [89]. Furthermore, dietary supplementation with 150 mg/kg of bisdemethoxycurcumin (a curcuminoid) demonstrated a positive effect against LPS-induced small intestinal oxidative damage by upregulating antioxidant-related genes such as CAT, γ-GCLc, Nrf2, HO-1, and NQO1 [90]. Despite the negative alterations caused by LPS challenge on the intestinal barrier function and inflammation in broilers, these effects have been found to be significantly restored with the supplementation of 400 mg/kg of resveratrol, which leads to the downregulation of pro-inflammatory cytokines and to the upregulation of genes related to the intestinal barrier, thereby ultimately improving immune function damage and alleviating intestinal inflammation [91].

In Hy-Line brown laying hens, SeMet (selenomethionine) has been shown to alleviate the eggshell gland cell necroptosis-mediated inflammation induced by LPS via regulating the Keap1/Nrf2/HO-1 pathway [92].

Moreover, in the context of mycotoxin exposure, the liver mitochondrial oxidative injury induced by aflatoxin B1 in broilers could be alleviated by a supplementation with lycopene, which would lead to the upregulation of the mRNA expression levels of hepatic MnSOD, TrxR2, and PGC-1α [93]. Additionally, a supplementation with taraxasterol has proven effective in alleviating aflatoxin B1-induced liver damage, which is achieved by regulating oxidative stress through the Keap1/Nrf2 signaling pathway, apoptosis, and autophagy [94]. Furthermore, selenium-enriched yeast supplementation has been found to relieve the damage caused by ochratoxin-A, which leads to an improvement in the inflammatory response via TLR4/MYD88 (toll-like receptor 4/myeloid differentiation factor 88) signaling pathway and to the protection of the intestinal barrier via the regulation of related genes like claudin-1, occludin, and ZO-1 [95]. The intestinal toxicity caused by ochratoxin-A is alleviated with the supplementation of selenium-enriched yeast via the activation of Nrf2 and inhibition of NF-κB [96]. In the case of AFB1 exposure, the expression levels of antioxidant genes (SOD, CAT, GPx1, and GST) are low while those of mitochondrial-apoptosis-associated genes (Bax, caspase-9, caspase-3, p53, and cytochrome c) are high. However, supplementation with 250 mg/kg of proanthocyanidins reverses these expression levels, as well as the upregulated antioxidant genes and downregulated genes associated with apoptosis, thereby significantly modulating the antioxidant defense system and apoptosis [97]. Furthermore, a novel antioxidant insight into myricetin revealed that broilers that have been infected with *Eimeria* spp. and supplemented with myricetin exhibited higher expression levels of genes related to antioxidant defense and anti-inflammatory activity. Additionally, they demonstrated higher expressions of peptides, thereby conferring a direct defense against microbial invasion (AvBD6 and AvBD612), which—in turn—indicates the promising antioxidant role of myricetin [98]. Table 1 provides more details on the studies mentioned above.

## 6. Conclusions

In conclusion, the supplemental administration of natural antioxidants influenced the expression of genes, which led to positive effects on broiler health by maintaining an optimal redox balance and improving their antioxidant defense and stress responses. This was achieved by regulating the inflammatory response and apoptosis, as well as by also improving lipid metabolism and the state of the intestinal barrier.

The reviewed studies can be divided into two categories. In the first category, there were studies that examined the action of natural antioxidant compounds in relation to the amount of their administration, while the second category included studies that, in addition to the natural compound, also used some type of stress factor (i.e., thermal stress, aflatoxin attack, lipopolysaccharides, etc.) and then examined their effect on gene expression separately, as well as in combination.

In the first category, the studies showed the improvement of antioxidant defense through activations of the transcription factor Nrf2 and increases in the genes that regulate the expression of antioxidant enzymes (e.g., SOD, GPx, CAT, and GCL) and detoxification enzymes (e.g., HO-1, NQO1, and GST). Many of the tested compounds showed an improvement in fatty acid metabolism by upregulating the expression of genes related to lipolysis (e.g., PPARα, ATGL, and CPT-I) and downregulating those related to lipogenesis (e.g., LXRα, SREBP-1c, FAS, and ACC). This regulation was mainly due to the activation of the AMPK pathway.

In the second set of studies, natural antioxidants were able to reverse some of the harmful effects caused by each stressor. When chickens were exposed to higher ambient temperatures, the expressions of genes related to the oxidative stress response and heat stress response (e.g., HSP27 and HSP70) were increased. However, their expressions were limited to the groups that were also fed with the natural antioxidant. As oxidative stress is associated with the inflammatory response, the subsequent activation of NF-κB resulted in the high expression of pro-inflammatory cytokines (e.g., IL-1β and IL-6) and to the limited expression of anti-inflammatory ones (e.g., IL-4 and IL -10) with the suppression of Nrf2 and to the reduced expression of the genes it regulates. Both lipopolysaccharide and mycotoxin challenges negatively affected the expression of the genes related to the state of the intestinal barrier (e.g., MUC2, ZO-1, occludin, and claudin-1); meanwhile, in the case of mycotoxins, a high expression of genes inducing apoptosis was found (e.g., Bax, caspase-3, and caspase-9). In the groups that had been supplemented with some natural antioxidant, the regulation of the above was reversed, whereby the intestinal barrier was kept intact and apoptosis was limited.

A diverse array of natural antioxidants exists; however, a predominant tendency in research involves the utilization of commonly recognized antioxidants. Further investigations incorporating novel antioxidants are warranted. Roots, seeds, leaves, and byproducts offer a cost-effective and easily accessible option. However, their varying concentrations in each recorded antioxidant may introduce variability, thus potentially influencing the ultimate outcome.

Exploring combinations of various commercially available natural antioxidants in trials would be of interest, as would the introduction of ‘nanocarriers’, which may impact costs but simultaneously enhance bioavailability. This, in turn, would allow a significant reduction in the dosages of natural antioxidants.

Microbiota, as well as immune and antioxidant defense system interactions, under eubiosis and dysbiosis conditions need to be elucidated in future studies since this was out of the scope of the present study. Given that studies in humans have revealed this link between microbiota, immune, and antioxidant systems, it is possible that similar coexistence might be true for broilers. For example, in ulcerative colitis patients, IgG interacts with gut macrophages expressing Fc-gamma receptor, which results in the induction of the NLRP3 inflammasome—a critical component of the innate immune system—and reactive oxygen species (which, in turn, stimulate the pro-inflammatory cytokine IL1b production [99,100]). In the present review, the valuable role of natural antioxidants in improving the productivity and health of broilers has been demonstrated in dose-dependent trials or in those trials that demonstrated the coexistence of antioxidants and pro-oxidants. In depth clarification of the mode of action and optimal dietary inclusion level of antioxidants also need to be addressed in future studies.

## Figures and Tables

**Table 1:** Literature review of the dietary administration of natural antioxidants and their effects on gene expression in broilers.

Natural Antioxidant/Dosage/Type of Stress Factor	Oxidative Stress Response	Inflammatory Response/Apoptosis	Metabolism/Intestinal Barrier	Conclusions	Reference
Rutin (0, 200, or 400 mg/kg)	↑Nrf2 (400 mg/kg) and ↑CAT (200 mg/kg)	-	↓AMPKα, ↑FADS1, ACC (200 and 400 mg/kg), ↑PPARγ, FAS, and ELOVL7 (400 mg/kg)	Dietary rutin supplementation appears to alter the fatty acid profile and metabolism by regulating the expression levels of the genes related to lipid metabolism	[73]
Lycopene (LYC: 10, 20, or 30 mg/kg)	↑Nrf2, NQO1, HO-1, and SOD2 (higher expression levels in the 30 mg/kg, 21st day); ↑Nrf2, SOD 2, and NQO1 (higher expression levels in the 30 mg/kg, 42nd day)	-	-	The supplementation with 30 mg/kg of LYC effectively improved the gene expression in the Keap1-Nrf2 signaling pathway. The researchers suggested an increase in the amount of LYC in the early stages of development	[66]
Rutin (0, 250, 500, or 1000 mg/kg)	↑Nrf2 and NQO1 (500 and 1000 mg/kg); ↑HO-1 (500 mg/kg); and ↑SOD with ↑ levels of rutin	↓BAX in jejunal mucosa (250 and 500 mg/kg); ↓NF-κB (500 mg/kg); ↓TNF-α (500 and 1000 mg/kg); and linear and quadratic ↓IL-2 (dose-dependent)	↑ZO-1 (500 and 1000 mg/kg), and ↑CLN and CLDN2 (500 mg/kg)	Dietary rutin supplementation improved jejunal morphology and enhanced intestinal barrier function through the inhibition of NF-κB and the activation of the Nrf2/HO-1 pathway. Optimum dose: 500 mg/kg	[68]
Magnolol (MAG: 100, 200, 300, or 400 mg/kg)	↑Nrf2, NQO1, HO-1, GCLC, and SOD (200 and 400 mg/kg, 28th day); ↑GCLC (significant upregulation with 100 and 400 mg/kg); ↑GST and SOD (100 mg/kg and 200 mg/kg, 51st day); and ↑Nrf2, NQO1, HO-1, GST, GCLM, and SOD (300 mg/kg)	-	-	Dietary MAG supplementation enhanced the oxidative stability by activating the Nrf2 pathway, which led to an increase in the expression of related genes such as NQO1, HO-1, and GSH	[65]
Genistein (0, 50, 100, or 150 mg/kg)	-	-	↓LXRα, SREBP-1c, FAS, and ACC (100 and 150 mg/kg); ↑PPARα, ATGL, and CPT-I (100 and 150 mg/kg); ↑FOXO1, ERβ, ↑SIRT1, AMPKα, and PGC-1a (100 and 150 mg/kg)	Dietary genistein supplementation has led to a reduction in abdominal fat deposition by downregulating the expression levels of genes associated with lipogenesis (LXRα, SREBP-1c, FAS, and ACC) and upregulating the expression levels of those associated with lipolysis (PPARα, ATGL, and CPT-I). Researchers have possibly attributed these results to the activation of the AMPK-SIRT1/PGC-1a pathway	[70]
Hesperidin (E1: 0.75 or Ε2: 1.5 g/kg feed) + Naringin (Ν1: 0.75 or Ν2: 1.5 g/kg feed)	Ν(+): ↑GSR (positive linear dose–response increase between the N1 and N2 groups)	-	Ν(+): ↑PPARα (positive linear dose–response increase between the two groups with a significant increase in the expression in N2 compared to N1) and ACOX1 (significantly increased in Ν1); Ν(+) ή Ε(+): ↑FASN (significant linear dose–response); and Ε1: ↑ADIPOQ	Supplementation with naringin significantly increased the expression levels of PPARα and ACOX1, i.e., the genes involved in the β-oxidation of fatty acids in the liver. Both hesperidin and naringin supplementation led to an increase in the FASN gene expression in breast muscles, which was perhaps due to the lack of fatty acids in the diet and the need to meet these requirements in the muscles. Meanwhile, both hesperidin and naringin led to the oxidative stability of meat through the regulation of the GSR gene with a significant increase, mainly in naringin supplementation.	[72]
Quercetin (0, 0.2, 0.4, or 0.6 g/kg)	-	-	↑AMPKγ (0.6 g/kg), ↑AMPKα1, AMPKα2, AMPKβ2 (0.2 g/kg), ↑PI3K (0.2 g/kg), ↑LKB1 (0.2, 0.4 and 0.6 g/kg), ↓ACC (0.4 and 0.6 g/kg), ↑CPT1 (0.6 g/kg), ↑PPARα (0.6 g/kg), ↓PPARγ, SREBP1 (0.2, 0.4 and 0.6 g/kg), and ↓HMGR (0.4 and 0.6 g/kg)	The supplementation with quercetin succeeded through increasing the expression levels of PI3K, PKB/ATK, and LKB1, as well as in activating the AMPK pathway and thus limiting adipogenesis and lipid synthesis by reducing the expression levels of SREBP1, PPARγ, and HMGR genes in the liver. The activation of the AMPK signaling pathway also prevented fatty acid intake, thus inducing lipolysis and oxidation by decreasing the expression levels of ACC and increasing that of CPT1 and PPARα, thereby contributing to the overall reduction in fat deposition in the livers of the broilers	[74]
Quercetin (200, 400, or 800 ppm)	↑SOD1 and GSH-Px (400 and 800 ppm)	-	↑GLUT2, PEPT1 (significantly increased in 800 ppm), and ↑FAS (significantly increased in 400 ppm)	In addition to increasing the expression levels of the antioxidant enzymes SOD1 and GSH-Px, quercetin supplementation upregulated the expression levels of nutrient transporter genes such as GLUT2 (sensor for glucose and its homeostasis), PEPT1 (key role in the absorption of small peptides), and FAS (contributes to the palmitoylation of the intestinal mucus barrier to protect the intestine from pathogens). Recommended doses are 200 and 400 ppm as 800 ppm did not affect the gut morphology as effectively as the other doses	[75]
Curcumin (0, 500, 1000, or 2000 mg/kg)	-	-	↓FAS, SREBP-1c, ↓ACLY, and ACC (2000 mg/kg); and ↑CPT-1 and PPARα (1000 mg/kg and 2000 mg/kg)	Curcumin supplementation contributed to the reduction in abdominal fat by decreasing the expression levels of the genes associated with lipogenesis (ACC, FAS, and SREBP-1c) and increasing the expression levels of the genes related to lipolysis (PPARα and CPT-I)	[71]
Resveratrol-loaded liposomal nanocarriers (Resv-Lipo NPs: 0, 50, 100, or 150 mg/kg) + Heat Stress (HS)	HS: ↑HSP70 and HSP90; and ↓SIRT1, SIRT3, SIRT7; Resv-Lipo NPs: ↑SOD, CAT, GSH-Px, Nrf2, and HO-1; ↑SIRT3, ↑SIRT1, and SIRT7 (100 mg/kg and 150 mg/kg); and ↓HSP70 and HSP90 (dose-dependent)	HS: ↑TNF-α and IL-6 Resv-Lipo NPs: ↑IL-10 (in a dose-dependent manner), ↓TNF-α, and IL-6	HS: ↓MyoD and mTOR; Resv-Lipo NPs: ↓myostatin, as well as ↑MyoD and mTOR (highest expression levels with 150 mg/kg)	Heat stress resulted in the upregulation of HSP70 and HSP90, which were restored to normal levels with the administration of 150 mg/kg of Resv-Lipo NP. The dietary supplementation with Resv-Lipo NPs could also alleviate the oxidative damage and modulate the expression levels of some myogenic regulatory factors, thus instigating muscle growth	[84]
Resveratrol (Resv: 400 mg/kg) + Heat Stress (HS)	HS: ↓Nrf2, HO-1, NQO1, GSH Px, and ↑Keap1; HS + Resv: ↑Nrf2, HO-1, NQO1 and ↓Keap1	-	-	Dietary Resv supplementation relieved the deterioration of meat quality by improving the muscle antioxidant capacity through the activation of Nrf2, thereby promoting its translocation within the nucleus and inducing the expression of genes related to antioxidant activity	[81]
Resveratrol (Resv: 500 mg/kg) + Heat Stress (HS)	HS + Resv: ↓HSP70	HS: ↑BCL-2, MDM2, and ↑ERK, IKB-α; HS + Resv: ↓BCL-2, Apaf-1, MDM2, and ↓NF-κB; and ERK, IKB-a, and p38 MAPK	-	Resv supplementation could reduce the inflammatory response by inhibiting the heat stress-induced activation of NF-κB, MAPK, and HSP70, as well as even prevent the activation of mitochondrial apoptotic pathways	[83]
Resveratrol (Resv: 400 mg/kg) + Heat Stress (HS)	HS: ↓Nrf2, SOD1, GPX, GST, and ↑Keap1; HS + Resv: ↑Nrf2, SOD1, GPX, GST, and ↓Keap1	-	-	Resv supplementation has proven to be an effective way through which to prevent the effects of heat stress by enhancing the intestinal antioxidant capacity through the activation of the Nrf2 signaling pathway	[82]
Astaxanthin (0, 10, 20, 40, or 80 mg/kg) + Heat Stress	↓HSP70, HSTF1, and ↑GST (quadratic changes); ↓GPX1, GR, and SOD1 (linear decrease)	↓JNK1 and TNF-α (linear decrease); ↑AKT1 and P38MAKP (quadratic changes)	↓SREBP1 (linear decrease); ↑DGAT2 (linear increase)	Dietary supplementation with astaxanthin affected the expression levels of the genes related to redox status (GST), heat stress (HSP70 and HSF1), inflammation (TNF-α), and lipid metabolism (SREBP1 and DGAT2)	[79]
Astaxanthin (0, 20, 40, or 80 mg/kg) + Heat Stress	↓HSP27 and HSP70 (40 or 80 mg/kg)	↓TNF-α (80 mg/kg); ↓IL-6 (40 or 80 mg/kg)	-	The decrease in HSP expression levels that came with astaxanthin supplementation was due to its antioxidant activity, which resulted in a reduction in the negative effects of heat stress and led to a reduction in heat stress-induced inflammation (TNF-α and IL-6)	[80]
Quercetin (Q: 200, 400, or 800 ppm) + Oxidized oil (Ox)	Ox(+): ↓TXN and HO-1; Ox(+)Q(+): ↑Nrf2, GCLM, CAT, SOD1, GPX2, GLRDX, TXN, and HO-1 (800 ppm); d ↓NOX2 (800 ppm)	Ox(+): ↓IL-8; Ox(+)Q(+): ↑IL-8	Ox(+): ↓MUC2 and ZO-1; Ox(+)Q(+): ↑MUC2, claudin-2 (800 ppm), and ↑ZO-1 (400 ppm)	Supplementation with 800 ppm of quercetin ameliorated the oxidative stress caused by oxidized oil, restored the redox balance, and strengthened the intestinal barrier	[78]
Squalene (SQ: 1.0 g/kg) + Diquat (DQ: 20 mg/mL)	DQ(+): ↑Nrf2, GPX1, and ↓NQO1; DQ(+)SQ(+): ↓GPX1 and ↑NQO1	DQ(+): ↑Bax and CASP3; DQ(+)SQ(+): ↓Bax and CASP3	-	SQ supplementation improved the oxidative status and alleviated liver injury by regulating apoptosis, thus indicating its hepatoprotective effect	[76]
Pterostilbene (PT: 400 mg/kg) + Diquat (DQ)	DQ(+): ↓SOD1, ↓SIRT1, PGC1a, and NRF1; PT(+): ↑NrF2, HO-1, SOD1, GSTA2, γ-GCLc, ↑SIRT1, and TFAM	PT(-)DQ(+): ↓BCL2 and ↑CASP3; PT(+)DQ(+): ↓CASP3	-	PT supplementation through its effect on the expression levels of the genes related to oxidative stress and apoptosis proved its protective potential against hepatic damage	[77]
Protocatechuic Acid (PCA: 300 or 600 mg/kg) + Lipopolysaccharide (LPS)	LPS(+): ↓CAT, SOD1, and GPx-1; PCA(+): ↑SOD1, CAT, and GPx-1 (600 mg/kg)	-	LPS(+): ↓OCLN, ZO-1, JAM2, and MUC2; PCA(+): ↑OCLN, JAM2, and MUC2 (300 or 600 mg/kg)	Dietary supplementation with PCA was able to protect the intestinal health of broilers by alleviating mucosal damage and preserving the morphological structure of the intestine through the regulation of OCLN, JAM2, and MUC2 genes, while 600 mg/kg of PCA supplementation restored the activity of antioxidant enzymes by regulating the expressions of CAT, SOD1, and GPx-1	[89]
Resveratrol (Resv: 400 mg/kg) + Lipopolysaccharide (LPS: 0.5 mg/kg BW)	-	Resv(-)LPS(+): ↑TLR4, MyD88, NF-kB, IL-1β, IL-6, TNF-α, and ↓IL-10; Resv(+)LPS(-): ↓TLR4, NF-kB, IL-1β, IL-6, and TNF-α; and Resv(+)LPS(+): ↓TLR4, NF-kB, and TNF-α	Resv(-)LPS(+): ↓MUC2, ZO-1, occludin, and claudin-1; Resv(+)LPS(-): ↑MUC2, ZO-1, occludin, claudin-1; and Resv(+)LPS(+): ↑occludin	Supplementation with Resv significantly helped in alleviating the LPS-induced intestinal barrier damage and inflammation by upregulating the expression levels of MUC2, ZO-1, occludin, and claudin-1, as well as by reducing the expression of the TLR4/NF-kB pathway and inflammatory factors	[91]
Lutein (LU: 0, 20, or 40 mg/kg) + Lipopolysaccharide (LPS: 1 mg/kg BW)	-	LPS(+)LU(+): ↓TLR4, MyD88, NF-κΒ, IL-1β, IL-6, ↑IL-4 (20 and 40 mg/kg), and ↑IL-10 (higher expression in 40 mg/kg)	LPS(+)LU(+): ↑occludin, vlaudin-1, and ZO-1 (20 and 40 mg/kg)	Supplementation with mainly 40 mg/kg of LU restored the LPS-induced intestinal barrier function by regulating the expression levels of occludin, claudin-1, and ZO-1, as well as by taming inflammation through the inhibition of the TLR4/MyD88 pathway, which reduces the expression levels of pro-inflammatory cytokines (IL-1β and IL-6) and increases that of anti-inflammatory cytokines (IL-4 and IL-10)	[87]
Resveratrol (Resv: 400 mg/kg) + Lipopolysaccharide (LPS: 1 mg/kg BW)	-	Resv(-)LPS(+): ↑IL-1β, IL-8, IL-17, TNF-α, and ↓TGF-β; Resv(+)LPS(+): ↓IL-8, IL-6, IL-17, and TNF-α	Resv(-)LPS(+): ↓claudin-1, claudin-5, occludin, and ZO-1 Resv(+)LPS(+): ↑claudin-5, occludin, and ZO-1	Supplementation with Resv could improve the intestinal barrier function by increasing the expression levels of the genes associated with it, and it could also effectively alleviate the intestinal inflammation caused by LPS	[88]
Bisdemethoxycurcumin (BDC: 0 or 150 mg/kg) + Lipopolysaccharide (LPS)	BDC(-)LPS(+): ↓CAT, CuZnSOD, γ-GCLc, γ-GCLm, GSH-Px, GR, Nrf2, HO-1, and NQO1 (in jejunum and ileum); BDC(+)LPS(-): ↑CuZnSOD, γ-GCLc, Nrf2, HO-1, and NQO1 (in jejunum and ileum); ↑CAT and GR (in jejunum); BDC(+)LPS(+): significant ↑CAT, γ-GCLc, Nrf2, HO-1, NQO1 (in jejunum); and ↑γ-GCLc, Nrf2, and CuZn-SOD (in ileum)	-	-	Supplementation with BDC, a related compound of curcumin, demonstrated favorable protection against the oxidative damage that was caused by LPS in the small intestine by activating Nrf2 and upregulating the expression levels of the genes related to the antioxidant system	[90]
Taraxasterol (TAR: 25, 50, or 100 mg/kg BW) + Aflatoxin B_1_ (AFB1: 1 mg/kg)	AFB1(+)TAR(-): ↓Nrf2, NQO1, HO-1, and ↑Keap1; AFB1(+)TAR(+): ↑Nrf2 (100 mg/kg), ↑NQO1, and HO-1 (50 and 100 mg/kg); and ↓Keap1 (25, 50 and 100 mg/kg)	AFB1(+)TAR(-): ↓Bcl-2, ↑Bax, and caspase-3; ↑PI3K, AKT, and mTOR; AFB1(+)TAR(+): ↑Bcl-2 (25, 50 and 100 mg/kg), ↓Bax (50 and 100 mg/kg), and ↓caspase-3 (25, 50 and 100 mg/kg); and ↓PI3K, AKT, and mTOR (50 and 100 mg/kg)	AFB1(+)TAR(-): ↑CYP1A1 and CYP2A6; AFB1(+)TAR(+): ↓CYP2A6 (50 mg/kg); and ↓CYP1A1 and CYP2A6 (100 mg/kg)	TAR supplementation could alleviate the liver damage caused by AFB1, which inhibits oxidative stress, through regulation of the Keap1/Nrf2 signaling pathway. This would improve anti-apoptotic ability and restore the autophagy of hepatocytes by regulating the expression levels of Bcl-2, Bax, and caspase-3, as well as by inhibiting the PI3K/AKT/mTOR pathway	[94]
Lycopene (LYC: 200 mg/kg) + Aflatoxin B_1_ (AFB1: 100 μg/kg)	AFB1(+): ↓MnSOD, Trx2, TrxR2, Prx3, and PGC-1a; ↓NRF1 and TFAM; and AFB1(+)LYC(+): ↑MnSOD, TrxR2, PGC-1a, and ↑TFAM	-	-	LYC supplementation ameliorated AFB1-induced liver injury by upregulating the expression levels of the genes responsible for antioxidant defense and by maintaining mitochondrial biogenesis	[93]
Selenium-enriched Yeast (SY: 0.4 mg/kg/feed) + Ochratoxin-A (OTA: 50 μg/kg/BW)	-	(OTA): ↑NF-κB, TLR4, and MYD88; (OTA + SY): ↓NF-κB, TLR4, and MYD88	(OTA): ↓claudin-1, occludin, and ZO-1; (OTA + SY): ↑claudin-1 and occludin	The supplementation with SY alleviated the oxidative injury that was caused by OTA, and this was achieved through regulation of the TLR4/MYD88 pathway and inhibition of the NF-κB signaling pathway while protecting the intestinal barrier	[95]
Selenium-enriched Yeast (SY: 0.4 mg/kg/feed) + Ochratoxin-A (OTA: 50 μg/kg/BW)	(OTA): ↓Nrf2 and HO-1 (OTA + SY): ↑Nrf2 and HO-1	(OTA): ↑NF-κB, IL-1β, and TNF-α (OTA + SY): ↓NF-κB, IL-1β, and TNF-α	-	SY supplementation alleviated the intestinal toxicity that was caused by OTA through the activation of Nrf2 and inhibition of NF-κB	[96]
Proanthocyanidins (PC: 250 mg/kg) + Aflatoxin B_1_ (AFB1: 1 mg/kg)	PC(-)AFB1(+): ↓SOD, GST, CAT, and GPx1; PC(+)AFB1(+): ↑SOD, GST, CAT, and GPx1	PC(-)AFB1(+): ↑Bax, caspase-3, caspase-9, p53, cytochrome-C, and ↓Bcl-2; PC(+)AFB1(+): ↓Bax, caspase-3, caspase-9, p53, cytochrome-C, and ↑Bcl-2	-	PC supplementation could reverse the negative effect of AFB1 by reducing oxidative stress, thereby enhancing the antioxidant defense system and simultaneously inhibiting AFB1-induced apoptosis by regulating the expression levels of the genes associated with it	[97]
Myricetin (Myc: 200, 400, or 600 mg/kg) + *Eimeria* spp. (IC)	IC(+)Myc(-): ↓CAT, SOD, GSH-Px, HO-1, NQO1 (intestine and muscles), and ↑COX-2; IC(+)Myc(+): ↑CAT, SOD, GSH-Px, HO-1, NQO1 (↑expression with ↑dose), and ↓COX-2 (↓expression with ↑dose)	IC(+)Myc(-): ↑IL-1β, IL-6, TNF-α, ↓IL-10, AvBD6, and AvBD612; ↑CCL4, CCL20, and CXCL13; IC(+)Myc(+): ↓IL-1β, IL-6, TNF-α (in a dose-dependent manner),↑IL-10, AvBD6, AvBD612; and ↓CCL4, CCL20, and CXCL13 (higher levels of Myc led to more significant downregulation)	-	Supplementation with Myc improved the broiler’s response against *Eimeria* spp. by regulating the expression levels of the genes related to antioxidant defense (CAT, SOD, GSH-Px, HO-1, and NQO1) and the genes with anti-inflammatory (IL-10) and pro-inflammatory (IL-1β, IL-6, TNF-α) activity, as well as by decreasing the expression of chemotactic cytokines (CCL4, CCL20, and CXCL13) and increasing the expression of peptides that confer direct defense against microbial invasion (AvBD6 and AvBD612)	[98]

(↑: Increase in expression levels and ↓: decrease in expression levels).

## Data Availability

Data are contained within this manuscript.
